# Phage display screening of therapeutic peptide for cancer targeting and therapy

**DOI:** 10.1007/s13238-019-0639-7

**Published:** 2019-05-28

**Authors:** Phei Er Saw, Er-Wei Song

**Affiliations:** 1 Guangdong Provincial Key Laboratory of Malignant Tumor Epigenetics and Gene Regulation, Sun Yat-sen Memorial Hospital, Sun Yat-sen University, Guangzhou, 510120 China; 2 Breast Tumor Center, Sun Yat-sen Memorial Hospital, Sun Yat-sen University, Guangzhou, 510120 China

**Keywords:** phage display, tumor targeting peptide, tumor vasculature, tumor microenvironment, tumor stromal cells, over-expressed receptor

## Abstract

Recently, phage display technology has been announced as the recipient of Nobel Prize in Chemistry 2018. Phage display technique allows high affinity target-binding peptides to be selected from a complex mixture pool of billions of displayed peptides on phage in a combinatorial library and could be further enriched through the biopanning process; proving to be a powerful technique in the screening of peptide with high affinity and selectivity. In this review, we will first discuss the modifications in phage display techniques used to isolate various cancer-specific ligands by *in situ*, *in vitro*, *in vivo*, and *ex vivo* screening methods. We will then discuss prominent examples of solid tumor targeting-peptides; namely peptide targeting tumor vasculature, tumor microenvironment (TME) and over-expressed receptors on cancer cells identified through phage display screening. We will also discuss the current challenges and future outlook for targeting peptide-based therapeutics in the clinics.

## Introduction

Peptides are 2-dimensional, linear chains of amino acids, which are usually short (less than 50 AA) in length (Hayashi et al., 2012). They are either designed by rational computing methods or phage display screening to obtain peptides that binds with high specificity to the target of interest, with a possibility of modulating the target (Marqus et al., 2017). Compared to antibodies (~150 kDa), peptides are relatively small (~3–5 kDa) and therefore easy to synthesize and modified (Boohaker et al., 2012), have higher cell membrane penetration, and possess less immunogenicity. In cancer therapy, these peptides can be used as a targeting ligand assisting specific delivery of cytotoxic drug specifically into the tumor vasculature, tumor microenvironment or into the cancer cells. On the other hand, peptides could also be delivered intracellularly to target cancer specific upregulated transcription factors, oncogenes or enzymes (Jyothi, 2012; Marqus et al., 2017). The general comparison between antibody and peptide are summarized in Table 1.

**Table 1:** The advantages of peptide as compared to antibody

	Antibody	Peptide
Size	150 kDa	3–5 kDa
Affinity (*K*_D_)	pmol/L–nmol/L	pmol/L–nmol/L
Immune response	Little	Little
Tissue penetration	Low	High
Intracellular target	No	Yes
Research cost	High	Low
Production cost	High	Low
Developing speed	Months–years	Months
Patent barriers	High	Minimal

Herein, we will review the utilization of phage display biopanning with modifications gearing towards *in situ*, *in vitro*, *in vivo*, *ex vivo* and in human application for high affinity peptide screening. We will also provide a comprehensive discussion on the latest discovery of tumor targeting-peptides; namely the peptides targeting (1) tumor vasculature, (2) tumor microenvironment (TME) and (3) over-expressed receptors on cancer cells.

### Phage display technology and biopanning strategies

In 1985, George Smith first described phage display by demonstrating the ability of a filamentous phage to display peptide by fusing the library of peptide sequence into the virus’s capsid protein (Smith, 1985). Since the peptide was displayed on the viral surface, selection could be done to isolate those with the highest binding affinity towards a target. In the same year, Geroge Pieczenik filed a patent also describing the generation of phage display libraries in detail (US patent, 5866363). However, the application of this technology was pioneered by Greg Winter and his colleagues at the Scripps Research Institute for display of proteins (specifically antibodies) for therapeutic protein engineering. Due to their contribution in phage display technique development and the enormous implication of phage display technology, Smith and Winter were both awarded a quarter share of the 2018 Nobel Prize in chemistry, while the other half was awarded to Frances Arnold.

**Figure 1:**
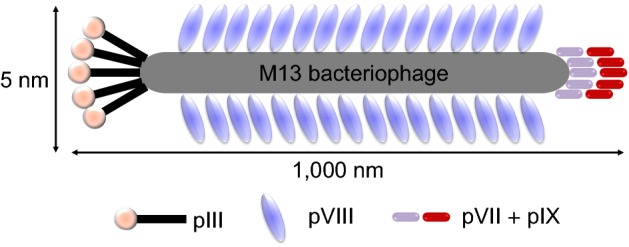
**A typical representation of M13 phage with about 1,000 nm in length and 5 nm wide**. The major coat proteins are pIII (green), pVIII (purple) and pVII + pIX complex (yellow + red)

Phage-display is a powerful technology for screening and isolating target specific peptides. This method utilizes bacteriophage to display foreign peptides or antibodies on their surface through insertion of the gene encoding the corresponding polypeptides into the phage genome. For display of foreign polypeptides on the bacteriophage, the desired DNA sequence is inserted into the M13 phage pIII or pVIII gene (Fig. 1). The methodology using the major coat protein pVIII provides a multivalent display, however only short peptides (6–7 AA) could be displayed on pVIII gene. Therefore, most combinatorial libraries such as antibodies or proteins have been displayed using minor coat pIII. Since there could be only 3–5 copies of pIII protein per phage, this method limits the copy number but the length of foreign or synthetic polypeptides that can be expressed (Fig. 1).

**Figure 2:**
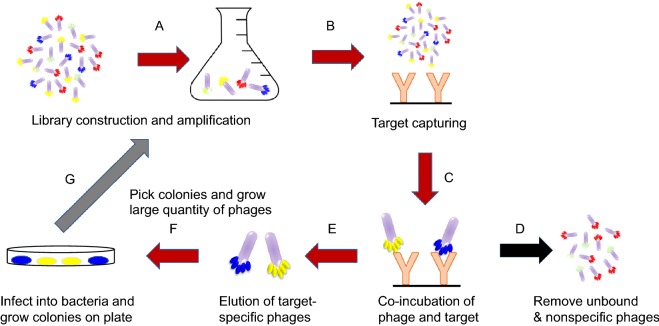
**The general scheme of phage display technique and biopanning selection of high affinity peptide**. Peptide-based library is first obtained either commercially or specifically designed to cater for specific needs of each experiment

The phage selection method, referred to as biopanning, is an affinity selection process that isolates target-binding molecules. As explained in Fig. 2, generally phage display based biopanning consists of five screening steps for selection of peptides. The first step is *“library construction* & *amplification”* where polypeptide-displayed phage libraries were constructed via cloning of combinatorial DNA sequence (Fig. 2A). This library will be amplified prior to biopanning (Fig. 2B). The second step is the *“target capturing step*”, in which the phage library is incubated with target molecule for a specific time to allow binding (Fig. 2C). The third step is to “*remove unbound & nonspecific phages*” by using repetitive washing to remove any unbound and non-target specific phages (Fig. 2D). The fourth step is the “*elution step*”, in which target-bound phages are separated after a short incubation with low pH buffer or by competitive elution (Fig. 2E). Finally, in the fifth step “*infection stage*”, the eluted phages are infected in bacteria to amplify selected phages, making a new and more selective phage library that should be applied in a next round of biopanning (Fig. 2F and 2G).

In general, three to five rounds of biopanning are necessary to isolate specific and high affinity peptide binders. Nonspecific phages are removed and phages with high affinity for the target are isolated by increasing the stringency in each round of biopanning by increasing the number of washing and decreasing the amount of target molecule. At the end of biopanning, phage ELISA and DNA sequencing are used for identification of individually specific phage with high affinity to target.

### Ample research to isolate high affinity peptide by phage display screening

Although *in situ* phage display screening using immobilized antigen is capable of generating high affinity and specificity peptide (Kim et al., 2012b), to better mimic cellular and body condition, ample researches are being done on *in vitro*, *in vivo* (Liu et al., 2018), *ex vivo* (Sorensen and Kristensen, 2011) and even in cancer patient (Krag et al., 2006) screening for high affinity peptide in a heterogenous environment as this is a closer representation to their original condition.

#### Homogenous *in situ *screening

**Figure 3:**
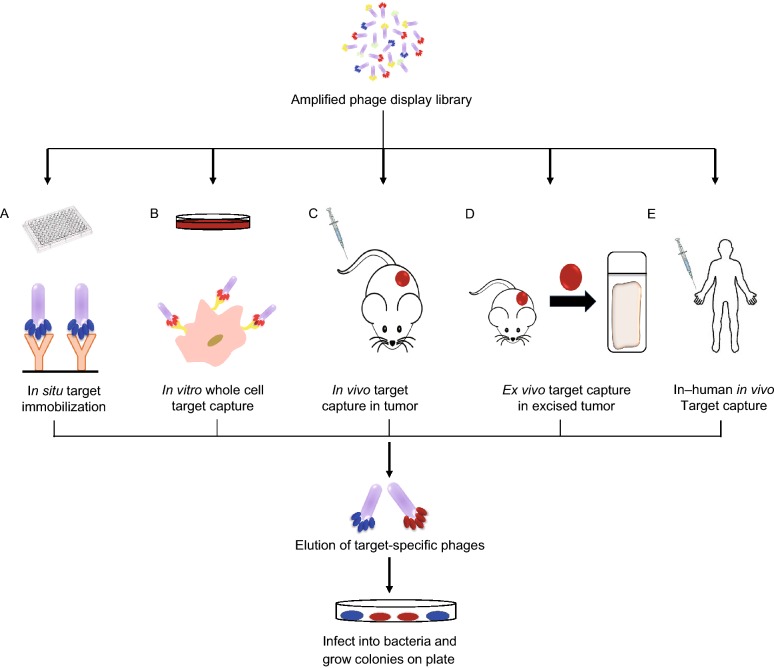
**Various approaches in capturing high affinity peptide through phage display screening**

Homogenous *in situ* screening requires only the specific target to be coated on a 96-well (Fig. 3A). A single target exposure guarantees the isolation of target-specific peptide, without external interference from non-specific binding. This method is also the easiest, as all experiments could be carried out without living system (i.e., cell culture, animal model, patient samples). The disadvantages of *in situ* screening includes the risk of non-specific binding of the isolated peptide when exposed to *in vitro* or *in vivo* system. In addition, the target is artificially coated onto the plate, which could be misrepresent the actual secondary structure of the target in a living system, therefore increases the risk of isolating a peptide that only binds to the receptor in this particular setting (Kim et al., 2012b).

#### *In vitro *cell screening

*In vitro* cell screening offers high-throughput approach for identifying multiple peptides that bind specifically to a single cell (i.e., cell lines or primary cells) and can be performed on adherent cells (live or fixed) (Fig. 3B). Advantages of using whole cell approach includes retaining their biological functions and activities, proper folding, 3-dimensional structure, receptor expression level and their association with neighboring proteins. Modified selection protocols could be used to isolate internalized peptides. Importantly, *in vitro* cell biopanning could identify novel cell surface receptors with unknown biological functions, which could be used to provide information on specific molecular changes (i.e., expression level of certain protein and their localization in normal vs. cancer cells) (Arap et al., 2002b; Zhao et al., 2007; Sun et al., 2012; Wu et al., 2016).

#### *In vivo *screening

By performing biopanning and selection in a living animal, organ-specific peptides could be isolated (Fig. 3C). Roushlati and co-workers first described *in vivo* phage display technology in 1996 (Pasqualini and Ruoslahti, 1996). For *in vivo* biopanning protocol, the biopanning selection is similar to that of the *in vitro* screening, the difference being the peptide phage library was introduced into the animal via systemic intravenous injection and allowed binding to occur within 1–2 h (as peptide-displayed phage is estimated to bound to target within 5–15 min (Laakkonen et al., 2002; Lee et al., 2007; Lo et al., 2008)), after which the animals will be perfused to remove unbound phages, sacrificed, and the desired organs will be collected and homogenized. Tissue-specific phage should increase after 3–5 rounds of biopanning (Rajotte et al., 1998; Lee et al., 2007; Chang et al., 2009). Through this approach, various types of tumor and malignant tissue vasculature have been identified (i.e., RGD-4C, NGR and GSL peptide (Koivunen et al., 1995; Pasqualini et al., 1997; Ruoslahti, 2000); detailed explanation below). One of the major pitfalls in using *in vivo* phage display technology is that the peptides may not be translated into human due to the differences of peptide binding between species (Wu et al., 2016).

#### *Ex vivo *screening

This method, first published in Nature in 2001, should only be applied to the selections of a specific rare cells in a heterogenous population (i.e., PBMCs in blood tumors) (Fig. 3D). Without sorting the cells, biopanning was performed on a glass slide containing the whole cell population. This method is advantageous for targeting a lower frequency of cells (<0.1% of the total population), as phages that binds non-selectively towards the other cells will be screened out. Once the phage was bound, UV irradiation was used so that the DNA of the phage particles on non-target cells is crosslinked by UV, while the phage on target cells were protected by a minute aluminum disc. Therefore, this method ensures that only non-crosslinked phage (target phage) were capable of replicating. The disadvantage of this method is that it is only optimized for antibody-based ligand selection, and thus not suitable for peptide selection. The yield of this method averages three antibodies per selection, which is very low compared to the other biopanning method (Sorensen and Kristensen, 2011).

#### In human screening

To diminish the compatibility of species difference between mice and human, phage display had been reported to be screened against human patients (Fig. 3E). The first in-human phage display screening was reported by Arap and colleagues in 2002. They reported a heptapeptide SMSIARL which could specifically home to prostate vasculature and exhibited 10–15 times more specificity to prostate compared to other organs (Arap et al., 2002b). Due to their success in proving safe usage of phage display in human, FDA approved similar techniques to be used by Krag and colleagues to screen tumor-specific peptide via phage display screening in terminal stage cancer patients (Krag et al., 2006).

## Tumor targeting peptide

Tumor targeting peptide is a powerful tool that could be used in cancer diagnosis and treatment (Heppeler et al., 2000) as they have lower production cost and scale-up, easy to synthesize and yet they possess most if not all the merits of a targeting ligand: high affinity and specificity towards the target, with the advantage of high tumor penetration as compared to the large-sized antibody-based ligand (AlDeghaither et al., 2015). In the complexity of solid tumor, a peptide could be used to target the malfunctioned tumor vasculature, the dense extra-cellular matrix, tumor stromal cells, or overexpressed receptor on tumors. Herein, we will discuss some prominent examples of peptides identified through phage display biopanning techniques and their application in the biomedical field.

### Peptide targeting tumor-microenvironment (TME)

**Figure 4:**
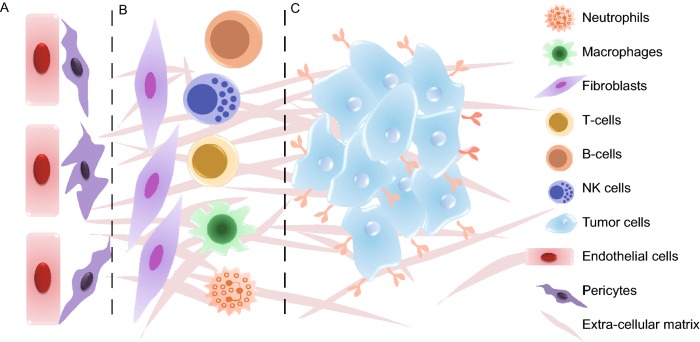
**Major components in the TME**. (A) tumor vasculature components and extra-cellular matrix, (B) tumor stromal cells and (C) over-expressed receptors on tumor cells

Tumor microenvironment (TME) is a complex plethora of multiple components including tumor-associated vasculature, extra-cellular matrix, cancer associated fibroblast, tumor associated macrophages, immune cells (neutrophils, NK cells, T cells, B cells) and tumor cells (Binnewies et al., 2018) (Fig. 4). Often, these cells transformed into tumor-like phenotype as tumor progresses. For example, most tumor resident macrophages are M2-like (pro-tumoral) which means they are programmed to assist in tumor growth rather than having an M1-like (anti-tumoral) phenotype (Mantovani et al., 2017). These changes could be brought forth by constant communication with the other components in the TME through autocrine or paracrine manner. Therefore, by identifying peptide specific to these TME targets could generate drugs homing to TME that could efficiently normalize, modulate or disrupt the TME components. There are three points of intervention, namely (i) targeting tumor vasculature, (ii) targeting extra-cellular matrix, (iii) targeting tumor stromal cells (macrophages, cancer associated fibroblasts etc.).

#### Peptide targeting tumor vasculature

Angiogenesis is an event of the formation of new blood vessels and is vital in the event of tumor growth and progression. Due to the continuous formation of new blood vessels to feed the tumor, a hyper-vascular tumor could grow beyond the size of millimeter in diameter (Bergers et al., 1999). Therefore, stopping a tumor’s blood supply can dramatically reduce the tumor growth, and in some cases, even resulted in total tumor eradication (Ferrara and Alitalo, 1999; O’Reilly et al., 1999). The morphology of tumor vasculature is very different from normal tissue vasculature. Due to the on-going angiogenesis, tumor vasculatures consistently express angiogenic marker at high concentration (i.e., integrins, VEGFR) and are usually tortuous (Bergers et al., 1999), with pronounced hypoxic region. Tumor vasculatures are also “leaky” in nature and this might be related to pericyte deficiency (Ruoslahti, 2000), therefore Folkman hypothesized that angiogenesis inhibition could be used to treat solid tumors (Folkman, 1971).

#### Peptide targeting tumor endothelial cells (EC)

The peptides that home to tumor vasculature may also be useful in targeting therapies specifically to tumors. Tumors are critically dependent on blood supply; therefore, blocking or eliminating that supply can profoundly suppress tumor growth (Denekamp, 1993; Hanahan and Folkman, 1996; Bergers et al., 1999; Jain, 2001). Since blood vessels are easily accessible through IV administration, and they do not readily acquire mutations as cancer cells that leads to drug resistance (Kerbel, 1991; Boehm et al., 1997), targeting tumor ECs could be a promising approach for targeted drug delivery.

A classic example of vasculature targeting peptide is none other than the “RGD” peptide. Rouslahti and colleagues first isolated this peptide by phage display *in vivo* in the form of cyclic peptide CDCRGDCFC (RGD-4C). This peptide has been validated to selectively binds αvβ3 and αvβ5 integrins (Koivunen et al., 1995); and have shown to home to the vasculature of tumors (Pasqualini et al., 1997). Interestingly, RGD domain is also vital for the binding of vitronectin and fibronectin and to integrins, although it is now known that these molecules bind to different subset of integrin (Ruoslahti, 2003).

Arap et al. also developed a set of cyclic peptide CNGRC sharing “NGR” motifs (Arap et al., 1998). These peptides have been shown to bind to tumor vasculatures in breast carcinoma, melanoma and Kaposi’s sarcoma (Pasqualini et al., 1997; Arap et al., 1998; Pasqualini et al., 2000). Subsequently, many other publications followed, describing the isolation of tumor vasculature related targeting peptides (Table 2) (Landon and Deutscher, 2003; Zurita et al., 2003; Ruoslahti, 2004; Kelly et al., 2005; Su et al., 2005).

**Table 2:** Peptide targeting TME and TME stromal cells

Target	Peptide sequence	Peptide affinity (*K*_d_)/LC_50_/IC_50_	Reference
Endothelium/av integrin	ACDCRGDCFCG (‘‘RGD’’ motif)	LC_50_~10 mmol/L	(Koivunen et al., 1995)
Endothelium expressing aminopeptidase N/CD13	CNGRC	LC_50_~34–481 mmol/L	(Pasqualini et al., 2000)
Breast endothelium/amino peptidase P	CPGPEGAGC	ND	(Essler and Ruoslahti, 2002)
Prostate endothelium	SMSIARL	ND	(Arap et al., 2002a)
Lung endothelium (membrane dipeptidase)	CGFECVRQCPERC	ND	
Skin endothelium	CVALCREACGEGC	ND	
MMP9	CRRHWGFEFC	IC_50_~10 mmol/L	(Ndinguri et al., 2012)
MMP2	CTTHWGFTLC	IC_50_~10 mmol/L	
Transmembrane chondroitin sulfate proteoglycan NG2	TAASGVRSMH	ND	(Burg et al., 1999)
	LTLRWVGLMS	ND	
Tumor-associated FN	CTVRTSADC	*K*_d_~11 µmol/L	(Han et al., 2015)
	HCSSAVGSWTWENGKWTWKGIIRLEQ	*K*_d_~65 nmol/L	(Kim et al., 2012b)
Tenascin C	FHKHKSPALSPV	4.58 ± 1.4 µmol/L	(Kim et al., 2012a)
Tumor associated macrophages (TAMs)	YEQDPWGVKWWY	ND	(Cieslewicz et al., 2013)
Cancer associated fibroblasts (CAFs)	HTTIPKV	MC = 0.70	(Brinton et al., 2016)
	APPIMSV	MC = 0.74	
Urokinase plasminogen activator (uPA) receptor (uPAR)	AEPMPHSLNFSQYLWYT	ND	(Landon and Deutscher, 2003)
	LWXXAr (Ar = Y, W, F, H) XFXXYLW	IC_50_~0.01–10 mmol/L	(Goodson et al., 1994)

#### Peptide targeting MMPs

Matrix metalloproteinases (MMPs) family is among the molecules that are upregulated in tumor microenvironment, and has been known to be functionally important in angiogenesis (Koivunen et al., 1999). Not only that, MMPs are also involved in increasing cell motility and invasiveness (Birkedal-Hansen, 1995). Although MMPs are secreted proteins, they are able to mediate phage homing. This might be due to the binding of MMP-2 and MMP-9 to αvβ3 integrin (Brooks et al., 1996), thus forming a complex that is stable enough for the binding of phage. Apparently, the complex is stable enough for strong binding of the phage to the MMP. Interestingly, the selected phage bound to MMP-2 and MMP-9 also specifically homes to tumor vasculature (Koivunen et al., 1999), indicating that (i) that one, or both, of these MMPs is specifically expressed in tumor vasculature and (ii) they are available for phage binding from the circulation. Multiple peptides inhibiting MMP families have been isolated through phage display screening. Their sequence, activities and function are summarized in Table 2 (Ujula et al., 2010; Ndinguri et al., 2012).

#### Peptide targeting pericytes of angiogenic vessels

Pericytes secrete growth factors that stimulate EC proliferation. Pericytes also secrete proteases to modulate the surrounding ECM and guide EC migration (Gerhardt and Betsholtz, 2003; Armulik et al., 2005; Saunders et al., 2006; Stapor et al., 2014). Recently, more researches are pointing towards the importance of pericyte coverage in vessel remodeling, maturation, and stabilization (Ribeiro and Okamoto, 2015). Therefore, pericyte might be the overlooked player in angiogenesis and should be given more emphasis in anti-tumor targeted therapy.

Several rounds of biopanning led Burg et al. to identify two decapeptides (TAASGVRSMH and LTLRWVGLMS) specific to a transmembrane chondroitin sulfate proteoglycan NG2, which is expressed in pericytes of angiogenic vessels (Schlingemann et al., 1990; Burg et al., 1999). These peptides specifically homed to tumor vasculature *in vivo* but not to tumor vasculature in NG2 knockout mice, indicating the specificity and targeting capability of these peptides (Burg et al., 1999). Although the role of NG2 in angiogenesis is still unclear, NG2 is a cell surface receptor for type-VI collagen and also binds to PDGF-A, which could potentially stimulate this growth factor (Nishiyama et al., 1996). As a component in pericyte, NG2 is undetectable in endothelial cells (Burg et al., 1999), therefore blocking NG2 represents a specific pericyte targeting.

#### Peptides targeting extra-cellular matrix (ECM)

The role of ECM components is now recognized as an important determinant in the growth and progression of solid tumors (Wernert, 1997; Pupa et al., 2002). ECM is extensively remodeled in tumor progression through 2 main processes: (i) neosynthesis of ECM components (i.e., alternative splicing mechanism of fibronectin to include EDA and EDB domain in malignant tumor fibronectin) and (ii) degradation of ECM by hydrolytic enzymes (e.g., proteases) that are produced, activated or induced by neoplastic cells, therefore become more permissive environment for tumor growth (Kaspar et al., 2006).

*Tumor-associated fibronectin* Fibronectin serves as a coordinator between cancer cells and ECM, and is involved in cancer cell survival, proliferation, invasion and metastasis (Wierzbicka-Patynowski and Schwarzbauer, 2003). One of the most extensive changes in ECM remodeling is the addition of extra-domain A and B (EDA and EDB), which are alternatively spliced-in during the synthesis of tumor-associated fibronectin. These domains are undetectable in healthy adult but has been found in high concentrations in malignant tumors. Clinical evidences indicated that tumor-associated FN (also termed oncofetal FN), is overexpressed in many malignant cancers, including breast cancer (Ioachim et al., 2002; Bae et al., 2013), prostate cancer (Suer et al., 1996; Albrecht et al., 1999), bladder cancer (Arnold et al., 2016), oral squamous cell carcinoma (Lyons et al., 2001), head and neck squamous cell carcinoma (Mhawech et al., 2005), colorectal cancer (Inufusa et al., 1995) and lung cancer (Khan et al., 2005), and upregulated FN expression has been correlated with poor prognosis of the patients. Therefore, tumor-associated FN represents an ideal target for solid tumor targeting.

Through *in situ* phage display technology, Kim et al. developed an EDB binding scaffold-like peptide termed APT_EDB_ (Kim et al., 2012b). APT_EDB_ consists of a stabilizing scaffold and two target-binding regions, mimicking the morphology of a DNA leucine zipper. Taking advantage of the synergistic three-dimensional structure for optimal binding, APT_EDB_ exhibited a high binding affinity (*K*_d_ ~65 nmol/L) to EDB and could be used as a targeting ligand to be conjugated to anti-cancer drugs for high tumor selectivity and reducing systemic toxicity (Kim et al., 2014; Kim et al., 2016), deliver biologics (i.e., oligonucleotides, siRNA and drugs) for solid tumor treatment (Saw et al., 2013; Saw et al., 2015; Saw et al., 2017) and to encapsulate superparamagnetic iron oxide particles for Magnetic Resonance Imaging of EDB over-expressing tumors (Park et al., 2012). In another study, Han et al. developed a cyclic nonapeptide (ZD2) with the sequence of CTVRTSADC that could be used for EDB specific targeting and imaging of prostate cancer. This linear peptide, which has *K*_d_ ~11 μmol/L binding affinity towards EDB, demonstrated excellent specific targeting to prostate cancer *in vivo* and could be utilized as an imaging agent for EDB-overexpressing prostate cancer (Han et al., 2015).

*Tenascin C* (*TNC*) TNC is a glycoprotein which forms a large structure body by assembling other ECM molecules and participates in cell adhesion, movement, permeation, survival, migration and differentiation (Chiquet-Ehrismann, 1990). As with tumor-associated FN, TNC is not usually expressed in normal cells except in immune tissues, such as bone marrow and thymus gland (Klein et al., 1993; Hemesath and Stefansson, 1994), but is specifically expressed in malignancy, inflammation and wound healing. It had been reported that the elevated expression of TNC depended on a malignancy in the tumor stroma of some malignancies, including oral cancer, sarcoma, breast cancer, and colon cancer, squamous cell carcinoma (Hindermann et al., 1999) chondrosarcoma (Ghert et al., 2001), breast cancer (Tsunoda et al., 2003) and colon cancers (Hanamura et al., 1997; Suzuki et al., 2017).

Kim et al. isolated a peptide that not only selectively bound to TNC in xenograft mouse tissue and patient tumors but also reduced TNC-induced cell rounding and migration. Due to the bulky size of TNC, they adopted two independent screening; the first using full-length TNC (expressed in eukaryotic cells) and the second using alternative spliced domain (expressed in bacteria). Out of a total of 35 clones, 19 had the same sequences (denoted peptide #1, FHKHKSPALSPV, 54.2% consensus) and another 13 clones were also identical (denoted peptide #2, FHKPFFPKGSAR, 37.1% consensus). The binding affinity of peptide #1 to TNC was 4.58 ± 1.4 µmol/L (Kim et al., 2012a).

### Peptide targeting tumor associated macrophages (TAMs)

High density of TAMs has been correlated to poor prognosis in several types of cancers, including brain, breast, ovarian and pancreatic cancers, where the majority of these TAMs express M2-like phenotype (Kurahara et al., 2011; Medrek et al., 2012; Colvin, 2014; Zhou et al., 2015). Therefore, M2-like TAMs have been exploited as therapeutic targets, and positive outcomes were shown in selective depletion of this macrophage subpopulations (Georgoudaki et al., 2016). Small molecules such as folic acid (targeting folate receptor β) and mannose (targeting mannose receptor) have been conjugated to drugs or carriers for macrophage targeting and drug delivery (Hashida et al., 2001; Low et al., 2008; Yu et al., 2013). However, these receptors are not macrophage specific and they are also expressed in other cell types for example, mannose receptors are also expressed in dendritic cells (Sallusto et al., 1995)). Folic acid also binds different isoforms of folate receptors on tumor cells and normal epithelial cells (Ross et al., 1994), therefore diminishing the specificity effect of the ligand. In 2012, Segers et al. reported a novel peptide that binds selectively to scavenger receptor-A on macrophages in atherosclerotic plaques. Nevertheless, it was found that this receptor is also expressed on dendritic cells (Segers et al., 2012). Therefore, M2-like macrophage-specific peptide should be screened and developed for clinical application.

Cieslewicz et al. polarized murine bone marrow-derived macrophages with either IFN-γ and LPS or with IL-4 to generate both M1 and M2 cells for biopanning. After three rounds of phage panning, highly selective M2 macrophage-binding peptides were identified, and this peptide binds preferentially to M2 cells. Sequencing of the 10 clones obtained above revealed two unique sequences: YEQDPWGVKWWY (denoted M2pep Phage, consensus 80%), and HLSWLPDVVYAW (consensus 20%). M2pep Phage demonstrated higher affinity and selectivity towards M2; 10.8-fold higher binding to M2 macrophages over scramble-M2pep, as well as 5.7-fold higher binding to M2 over M1 macrophages. Furthermore, after intravenous administration, M2pep Phage was able to selectively binds M2-like TAMs in mouse colon carcinoma tumors (Cieslewicz et al., 2013).

### Peptide targeting cancer associated fibroblasts (CAFs)

One of the dominant cell type in solid tumor is CAFs (Augsten, 2014). They are likely to be derived from the mesoderm and exhibited mesenchymal-like features (Kalluri and Weinberg, 2009). CAFs are often found in close vicinity or in direct contact with tumor cells (Kalluri and Weinberg, 2009). In normal condition, fibroblasts are likely to be quiescent or in resting state, yet became activated in response to growth factors, cytokines and mechanical stress (Kalluri and Weinberg, 2009; Rasanen and Vaheri, 2010; Shiga et al., 2015). Unlike tumor cells that presents diverse marker proteins on cell surface, CAFs selectively overexpressed certain proteins, such as fibroblast-activated protein-α (FAP-α) and α-smooth muscle actin (α-SMA) (Bhowmick et al., 2004; Kalluri and Zeisberg, 2006; Franco et al., 2010; Rasanen and Vaheri, 2010). Therefore, CAF targeting or responsive nanomaterial may be an efficient strategy to achieve improved antitumor efficacy.

Brinton et al. presented a new strategy for analysis by combining phage display and accompanying software: “PHage Analysis for Selective Targeted PEPtides” or PHASTpep, which they claimed to identify highly specific and selective peptides. Using this combination, they discovered and validated two peptide sequences (HTTIPKV and APPIMSV) targeted to pancreatic CAFs in mice. The Mander’s coefficient was high for both HTTIPKV (0.70) and APPIMSV (0.74) indicating phage clone binding to αSMA-positive CAFs *in vivo* (Brinton et al., 2016).

Urokinase plasminogen activator (uPA) receptor (uPAR) uPA is a serine protease largely produced in stromal fibroblast-like cells in melanoma, colon, breast, and prostate cancer. The uPA/uPAR interaction is important in early tumor development (i.e., cell adhesion and invasion). Goodson et al. isolated a uPAR specific peptide, AEPMPHSLNFSQYLWYT. This peptide was able to compete for binding of radiolabeled uPA fragment, therefore served as a potent antagonist for uPAR (Landon and Deutscher, 2003).

### Plausible targets for the development of tumor-targeting peptide

#### CD10+GPR77+ CAFs

Recently we demonstrated that CD10+GPR77+ CAFs specifically define a subset of CAF that correlated with chemoresistance and poor survival in breast and lung cancer patients. Mechanistically, the activation of CD10+GPR77+ CAFs was driven by the consistent NF-κB activation, which is maintained via GPR77 (C5a receptor) complement signaling. Furthermore, CD10+GPR77+ CAFs could lead to successful engraftment of patient-derived xenografts (PDXs), while blocking these CAFs with a neutralizing anti-GPR77 antibody inhibited tumor formation while restoring chemosensitivity of the tumor. Therefore, targeting the CD10+GPR77+ CAF subset could present an effective therapeutic strategy against solid tumors (Su et al., 2018).

#### CD146

Also known as melanoma cell adhesion molecule (MCAM), CD146 is a member of cell adhesion molecules of the immunoglobulin (Ig) superfamily (Lehmann et al., 1989). CD146 has been known to be involved in angiogenesis, tumor metastasis, lymphocyte activation, morphogenesis during development and tissue regeneration (Ouhtit et al., 2009; Wang and Yan, 2013; Ye et al., 2013). As CD146 is mainly expressed on ECs, CD146 is required for endothelial cell proliferation, migration and tube formation (Kang et al., 2006; Zheng et al., 2009), playing critical roles in angiogenesis (Yan et al., 2003; Chan et al., 2005; Harhouri et al., 2010; Kebir et al., 2010; Tu et al., 2015). To date, antibody-drug conjugate (ADC) targeting CD146 have been developed, therefore suggesting CD146 targeting could mitigate tumor growth and metastasis (Rouleau et al., 2015).

#### PITPNM3

Phosphatidylinositol transfer protein, membrane-associated 3 (PITPNM3), also known as Nir1, is essential in CCL18-induced chemotaxis through calcium influx. The function of PITPNM3 could be completely diminished by GPCR pathway inhibitor pretreatment or via pertussis toxin (PTX). We first demonstrated that PITPNM3 is abundantly expressed in breast cancer cells (Chen et al., 2011). In another independent research, He et al. revealed that PITPNM3 was also upregulated in hepatocellular carcinoma (HCC) cells and tissues. While the silencing of PITPNM3 significantly attenuated the invasiveness and metastatic ability of HCC cells, the upregulation of PITPNM3 increased HCC cell mobility. Mechanism wise, the inhibition of PITPNM3 suppressed the activation of Pyk2, FAK, and Src, and also impaired integrin clustering; indicating that PITPNM3 is a key player in cancer migration and invasion, therefore is a promising target in cancer therapy (C. He et al., 2014).

#### Transmembrane 4L six family member 1 (TM4SF1)

TM4SF1 was first discovered as a tumor cell antigen and could be specifically recognized by mouse monoclonal antibody L6 (Hellstrom et al., 1986b; Marken et al., 1992). TM4SF1 is expressed abundantly on many cancer cells (Hellstrom et al., 1986a; Hellstrom et al., 1986b), on tumor blood vessel endothelial cells (Shih et al., 2009). TM4SF1 is also associated with pathologic angiogenesis, targeting TM4SF1 would provide a dual anticancer mechanism: simultaneously targeting tumor cells and the tumor vasculature (secondary mechanism) (Visintin et al., 2015).

## Peptide targeting over-expressed receptors on tumor

In cancer treatment, overexpressed receptors are modulated by targeting agents such as antibodies, antibody fragments, peptides or small chemicals that could block their activities by directly binding these receptors, halting downstream mechanism therefore blocking cancer progression. Other approaches included exploiting receptor overexpression for the targeted delivery of anticancer drugs or biologically active molecules that are unable discriminate between cancer and normal cells. The ligand acts as their “eyes”, guiding them directly towards the overexpressed receptors on tumor cells, therefore specifically attacking malignant cells while sparing normal cells (Mendelsohn and Baselga, 2003).

**Figure 5:**
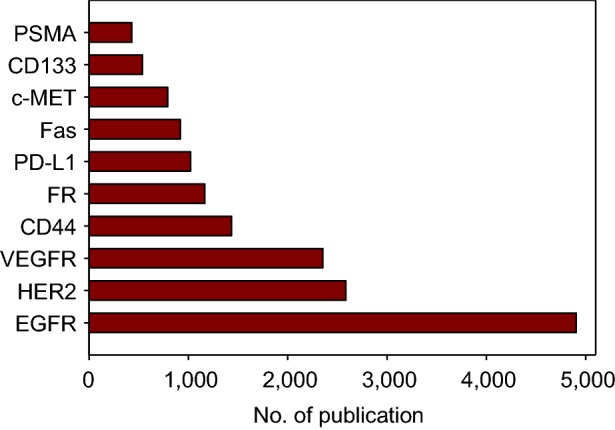
**Top-10 cancer-associated overexpressed receptors and their corresponding publications in PubMed until 2018**

In this section, we highlighted some prominent over-expressed receptors that have been widely used for cancer cell specific targeting. There is a myriad of targeting ligands that are currently known to be overexpressed in various cancer, differing in cancer types and subtypes, stages of cancer. It is quite a challenge to summarized all of these receptors in this review, therefore the selection was done on PubMed search with “receptor” and “targeting” filters. Figure 5 highlighted the Top-10 cancer-associated overexpressed receptors and their corresponding publications in PubMed until 2018. Comprehensive review of literature reveals that (with the exception of CD44 and Fas receptors), all other receptors had been used as targets for phage display biopanning and at least one peptide ligand has been developed for these receptors; which are highlighted in detail in the section below.

### ErbB family (EGFR & HER2)

In the ErbB family, there are four known members: ErbB1/EGFR/HER1 (only found in humans), ErbB2/HER2/Neu, ErbB3/HER3 and ErbB4/HER4 (Seshacharyulu et al., 2012). These receptors are transmembrane glycoproteins with molecular weights ranging from 170 to 185 kDa (Olayioye et al., 2000). EGFR are major contributors of a complex signaling cascade in cancer cells that modulates growth, signaling, differentiation, adhesion, migration and survival, therefore making EGFR an attractive candidate for anti-cancer targeting and therapy (Grandis and Sok, 2004). Specifically, EGFR has shown to play a key role in the development and growth of tumor cells, including cell proliferation and apoptosis (Wells, 1999).

In 2014, Wang, Zho and Joshi applied for an international patent (“Peptide reagents and methods for detection and targeting of dysplasia, early cancer and cancer”, Patent No. WO2016029125A1) for the screening and evaluation of EGFR-targeting peptide through *in situ* phage display screening, utilizing the PhD-7 heptapeptide random library and PhD-12 decapeptide random library provided by New England Biolab. The screening resulted in 17 EGFR-specific peptides: QRHKPRE, HAHRSWS, YLTMPTP, TYPISFM, KLPGWSG, IQSPHFF, YSIPKSS, SHRNRPRNTQPS, NRHKPREKTFTD, TAVPLKRSSVTI, GHTANRQPWPND, LSLTRTRHRNTR, RHRDTQNHRPTN, ARHRPKLPYTHT, KRPRTRNKDERR, SPMPQLSTLLTR and NHVHRMHATPAY; all showing selectivity and specificity towards EGFR. Nevertheless, the most prominent peptide sequences targeting EGFR so far are CMYIEALDKYAC (developed based on the structure of the natural EGF ligand) (Ai et al., 2011; Yang et al., 2016), YHWYGYTPQNVI (*K*_d_~22 nmol/L, also known as “GE11”) (Li et al., 2005; Song et al., 2008; Ren et al., 2015; Fan et al., 2016) and LARLLT (also known as “D4”) (Song et al., 2009; Ongarora et al., 2012; Lin and Kao, 2014; Fontenot et al., 2016), was also found to have high specificity towards EGFR, though D4 peptide was developed using a structural model, not through phage display technology.

HER2 is encoded by the ErbB-2 proto-oncogene. The growth and differentiation of cells are regulated by the intracellular domain of HER2 (Yarden and Sliwkowski, 2001; Cho et al., 2003), while the extracellular domain of HER2 interacts with HER family members to form heterodimer complex that facilitates signal transduction (Burstein, 2005). HER2 is a major contributor to breast cancer and about 20%–30% of BC cases are HER2 positive (Lee-Hoeflich et al., 2008; Li et al., 2016). HER2 genes could be amplified to nearly about 2 million receptors on the surface of tumor cells (Kallioniemi et al., 1992; Gutierrez and Schiff, 2011). Therefore, HER2 emerged as a trustworthy drug target when addressing HER2+ cancers (Baselga and Swain, 2009; Rimawi et al., 2015), ovarian (Menderes et al., 2017; Zanini et al., 2017) and gastric cancers (Ruschoff et al., 2012; Abrahao-Machado and Scapulatempo-Neto, 2016). Karasseva et al. described the selection of HER2-binding peptides using phage display. The peptide, KCCYSL bound to purified HER2 with a *K*_d_ of 30 mmol/L, and selectively bound to breast and prostate cancer cell lines, but not to normal cells (Karasseva et al., 2002). Houimel et al. isolated three linear peptides specific to HER2 (MARSGL, MARAKE, MSRTMS), and from here derived a humanized pentameric ‘‘peptabody’’ (Pab) molecules (fusion of linear peptide to an antibody-like tail). All three Pab bound to ErbB-2 with *K*_d_~6–16 nmol/L and inhibited HER2+ cancer cell growth and proliferation up to 40% (Houimel et al., 2001). Park et al. isolated bipodal peptide binder aptamer like peptide (aptide) specific to the extra-cellular domain of HER2 (APT_HER2_, *K*_d_ 89 mol/L). This APT_HER2_ was then conjugated onto superparamagnetic nanoparticles (SPION) for HER2-targeted specific magnetic resonance imaging (MRI) (Park et al., 2013).

### VEGFR

Vascular endothelial cell growth factor (VEGF) is a protein tyrosine kinase, and a well-known mediator of angiogenesis which is predominately mostly mediated by VEGF receptor family (VEGRR1, 2, 3; neurophilin 1) (Ferrara et al., 2003; Hoeben et al., 2004). Ample evidences now show that VEGFR family could be exploited as a potent therapeutic target in cancers. Often, the overexpression of VEGF and VEGFR are associated with invasion and metastasis in many malignancies (Prewett et al., 1999), including colorectal (Amaya et al., 1997; Duff et al., 2006), breast (Price et al., 2001; Ryden et al., 2003; Wulfing et al., 2005; Ghosh et al., 2008) and non-small cell lung cancers (Koukourakis et al., 2000; Kajita et al., 2001).

Giordano et al. introduced “Biopanning and Rapid Analysis of Selective Interactive Ligands” (termed BRASIL) as a new approach in the screening, selection and sorting of high affinity peptides. The novelty of this method lies in the additional step of cell-surface-binding peptides sorting based on differential centrifugation. Cell suspension was first incubated with phage in an aqueous upper phase, which will then be centrifuged through a non-miscible organic lower phase. Giordano and colleagues claimed that this single-step organic phase separation is faster, with enhanced sensitivity and specificity comparing to current methods that primarily rely on multiple washing steps or limiting dilution. Using HUVEC cells as a selection, they isolated 21 phage clones bound to starved HUVECs and to VEGF-stimulated HUVECs. Fourteen clones (67%) had a >150% enhancement (range, 1.5–8.7-fold; median, 2.2-fold) binding upon VEGF stimulation. Sequence alignment analysis of 34 clones randomly chosen from the selected phage revealed that 24 clones (70%) of the phage recovered through BRASIL selection had peptide motifs that could be perfectly mapped to sequences present in VEGF family members. They selected two peptides (CPQPRPLC and CNIRRQGC) for *in vitro* binding assay on VEGF receptor- 1 (VEGFR-1). One of the selected peptides, CPQPRPLC phage bound to VEGFR at over 1,000-fold enrichment as compared to control (Giordano et al., 2001).

### Folate receptor alpha (FRα)

Folate receptor alpha (FRα) is a 38-kDa glycoprotein, and is a receptor that binds to folates and mediates their intracellular transport(Henderson, 1990). FRα is significantly up-regulated in a many cancer such as ovarian cancer (OC), endometrial adenocarcinoma and non-small cell lung cancer (NSCLC) (Kane et al., 1988; Matsue et al., 1992; Kelemen, 2006). It is known that the expression of FRα is highly correlated with tumor grade, stage, malignancy and aggressiveness (Bueno et al., 2001; Hartmann et al., 2007), therefore suggesting that FRα is a promising target for tumor therapy and diagnosis.

Xing et al. reported a FRα specific 12-mer peptide C7 (MHTAPGWGYRLS, *K*_d_~0.3 μmol/L) isolated through four rounds of biopanning by using a Ph.D.-12 phage library displaying random dodecapeptides. The tumor targeting ability of C7 was confirmed in *in vivo* phage homing experiment and fluorescence imaging. C7 was accumulated at the site of tumor tissue, indicating that the peptide has the ability to target tumor tissue without phage environment, indicating the probability of using this peptide for FRα targeted therapy (Xing et al., 2018).

### PD-L1

Immune checkpoint inhibition has demonstrated significant success in cancer treatment in recent years, as host immune response could recover from tumor evasion (Pardoll, 2012). By evoking the host’s innate immune response, patients can potentially negate the tumor’s ability to resist targeted therapy, eliminating the need for continuous lines of therapy (Tumeh et al., 2014). One of particular interest is the interaction between programmed cell death receptor 1 (PD-1) and its ligand, programmed cell death ligand 1 (PD-L1) (Zou et al., 2016). PD-L1 expression allow tumor cells to go unrecognized by immune T-cells as foreign. The overexpression on PD-L1 on tumor cells would interact with the PD-1 on the T-cell surface, inhibiting the T-cell to destroy the foreign (tumor) cell (J. Naidoo et al., 2014) Overexpression of PD-L1 has been reported in many different tumor types, such as melanoma (40%–100%), NSCLC (35%–95%), glioblastoma (100%), ovarian cancer (33%–80%), and colorectal adenocarcinoma (53%) (Chen et al., 2012).

Recently, Li et al. used a random bacterial surface display library to screen and identify the PD-L1 binding peptides, and further enriched the peptide binding with PD-L1 with magnetic-activated cell sorting (MACS) and fluorescence-activated cell sorting (FACS). From the initial 5 × 10^6^ peptides library after one cycle of MACS, after eight cycles of FACS, the percentage of peptide in the sorting gate increased from 2.1% (40 nmol/L PD-L1) to 54.1% (10 nmol/L PD-L1). Sequencing of forty bacterial clones revealed nine different peptide sequences with the consensus sequence CWCWR, *K*_d_~95 nmol/L. The soluble peptides of the CWCWR sequence were synthesized, and the binding specificity was tested in PD-L1 high-expressing MDA-MB-231 and low-expressing MDA-MB-435 breast cancer cell lines (Li et al., 2018).

### c-MET

c-MET, also called tyrosine-protein kinase Met or hepatocyte growth factor receptor (HGFR), is a protein that is encoded by the MET gene. MET gene was discovered as a proto-oncogene more than two decades ago and it has been extensively studied (Cooper et al., 1984; Bottaro et al., 1991). Met could be activated via autocrine, paracrine, or genetic mutations that can lead to tumorigenesis, angiogenesis and metastasis (Rong et al., 1993; Rong et al., 1994; Takayama et al., 1997). Various studies have linked the overexpression of this C-Met-ligand-pair to most types of human solid tumors, including brain (Jung et al., 1994), breast (Altstock et al., 2000), ovary (Huntsman et al., 1999), thyroid (Di Renzo et al., 1992), pancreas (Ebert et al., 1994), stomach (Di Renzo et al., 1991), prostate (Humphrey et al., 1995) and nasopharyngeal carcinoma (Qian et al., 2002).

To isolate a specific c-Met-binding peptide, Zhao et al. screened for a Met-binding peptide (YLFSVHWPPLKA, *K*_d_~64.2 nmol/L), designated Met-pep1. Met-pep1 binds to Met on the cell surface and thus competed with HGF for Met binding. Interestingly, Met-pep1 is internalized by the cells after binding, and inhibited human leiomyosarcoma SK-LMS-1 proliferation *in vitro*. In SK-LMS-1 mouse xenograft model, tumor-homing of Met-pep1 was evident as early as 1 h post-injection and remained visible in some animals as late as 24 h post injection (Zhao et al., 2007), indicating that Met-pep1 could be used as a diagnostic agent or a therapeutic carrier in c-MET overexpressing tumors.

### CD133

CD133 is first identified as an antigenic marker for hematopoietic stem cells (Miraglia et al., 1997; Yin et al., 1997). CD133 is found to be expressed in several hematopoietic malignancies including acute myelogenous leukemia (Horn et al., 1999), chronic lymphocytic leukemia (Waller et al., 1999), and myelodysplastic syndromes (Green et al., 2000). Recently, CD133 has been reported to be overexpressed in several solid tumors including retinoblastoma (Hemmati et al., 2003), glioblastoma (Singh et al., 2003; Singh et al., 2004), prostate adenocarcinoma (Collins et al., 2005; Rizzo et al., 2005), kidney carcinoma (Florek et al., 2005), pancreatic cancer (Hermann et al., 2007) and colorectal cancers (O’Brien et al., 2007). Importantly, in glioblastoma and colorectal cancer, CD133-expressing cells are considered cancer stem cells (CSCs) as they mediate tumor initiation and metastasis (Singh et al., 2004; O’Brien et al., 2007; Ricci-Vitiani et al., 2007). These small population of CSCs are considered the tumor initiating cell population, and CSCs are often insensitive to chemotherapy and radiation treatment (Neuzil et al., 2007; Tang et al., 2007). Bao et al. showed that CD133+ glioma stem cells mediate radiation resistance in highly malignant gliomas (Bao et al., 2006). Therefore, targeting CD133+ would present an opportunity to eradicate tumor initiating cells, CSCs and tumor cells, also potentially drug-resistant cancer subpopulations (Smith et al., 2008).

Sun et al. identified a peptide binding specifically to mouse CD133, LS-7 (LQNAPRS, *K*_d_~ ND). Co-localization of LS-7 was seen with CD133+ cells but not CD133- cells. LS-7 significantly inhibited cell migration of colon and breast cancer cells. In mice, *in vivo* treatment of LS-7 homed with high specificity towards CD133+ cells indicating CD133 could be a potential target for anti-motility and anti-metastasis strategy especially in cancer stem cell therapy (Sun et al., 2012).

### Prostate-specific membrane antigen (PSMA)

PSMA is a 100 kDa type II transmembrane glycosylated protein and as the name implies, is overexpressed in nearly all prostate cancers cells, its expression is 100–1000 times higher in tumor tissues compared to normal tissues (Wright et al., 1995). The initial descriptions of an increase in PSMA expression in prostate cancer was associated with higher tumor grade with the presence of metastases (Bostwick et al., 1998; Sweat et al., 1998; Chang et al., 2001) suggesting that PSMA is a highly plausible target for PSMA-positive prostate cancer therapy and since has been adopted as a biomarker for diagnosis and imaging (Barve et al., 2014).

To screen for novel PSMA-specific peptide to be used as targeting ligands and targeted drug delivery to prostate cancer cells, Jin et al. identified PSMA-specific peptides through combinatorial phage display techniques. After five rounds of biopanning against recombinant human PSMA extracellular domain (ECD), GTI tripeptide was identified as the highest affinity peptides towards PSMA ECD, with *K*_d_ values of the GTI peptide to PSMA-positive LNCaP and C4-2 cells are 8.22 μmol/L and 8.91 μmol/L, respectively. Conjugation of GTI peptide with the proapoptotic peptide D(KLAKLAK)2 induced cell death in LNCaP cells. Also, GTI peptide shows the highest uptake in C4-2 xenografts, with minimal uptake in other organs (Jin et al., 2016).

## Scarcity of intracellular targeting peptide: a case study for APT_STAT3_

Most peptide therapeutics are peptides targeting intracellular checkpoints in tumor as these peptides could exert therapeutic effects *per se* via binding and inactive their targets. These peptides usually target transcription factor, enzymes or overexpressed oncogene that are not visible extracellularly. Oncogene-targeted therapeutic strategies have been shown to sensitize tumor cells to the effects of chemotherapy and radiotherapy, and act synergistically with the traditional chemo- and radiotherapeutics (Kumar et al., 1996; Milas et al., 2000; Yu and Hung, 2000; Argiris et al., 2004; Ropero et al., 2004). Nevertheless, compared to extracellular targeting peptide, publications related to intracellular targeting peptide in the suppression of oncogenes or transcription factor has not been on par, and this might be attributed to the inefficiency of the peptides to effectively cross the cellular membrane. However, if succeeded in overcoming this barrier, peptides could be much more effective than antibodies or their derivatives due to the absence of thiolated secondary structure, allowing peptides to retain their original secondary structure in exerting the targeting effect.

**Figure 6:**
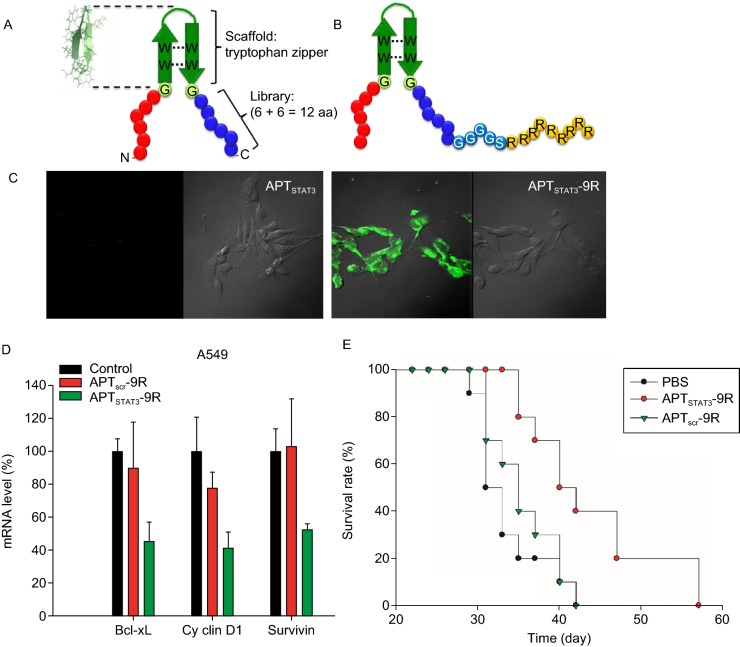
**Efficient intracellular delivery of peptides**. (A–C) APT_STAT3_ conjugated with cell penetrating peptide (CPP), 9R allowed high intracellular targeting of APT_STAT3_. (D) Unlike antibodies, the absence of disulfide bond in the secondary structure of peptide ensures that APT_STAT3_ remained biologically active in high glutathione (GSH) condition in the intracellular compartment. (E) treatment with APT_STAT3_-9R prolonged mice survival

STAT3 has received much attention for the important role it plays in signaling pathways linked to cancers (Yu et al., 2009). In cancer cells notably, STAT3 tends to be constitutively activated and had been associated with tumorigenesis and malignancy. Constant STAT3 activation leads to the production of a number of cytokines that regulate proliferation, angiogenesis, survival, and metastasis (Yu et al., 2007). Therefore, many research groups have tried to develop STAT3 inhibitors that can block upstream or downstream elements in the STAT3 signaling pathway (Benekli et al., 2009; Yue and Turkson, 2009). We previously reported an identification of STAT3-binding peptide (APT_STAT3_, *K*_d_ ~231 nmol/L). Conjugation of APT_STAT3_ with a cell-penetrating peptide 9R (APT_STAT3_-9R) was developed for enhanced cellular uptake. Not only APT_STAT3_-9R blocked STAT3 phosphorylation, they also reduced the expression of STAT downstream molecules in various types of cancer cells (melanoma, breast, lung, liver and brain cancer) Furthermore, intra-tumoral injection of APT_STAT3_-9R exerted potent antitumor activity in both xenograft and allograft tumor models. This study suggested a solid preclinical proof-of-concept for APT_STAT3_ as a powerful agent for STAT3 inhibition for targeting broad array of cancers with constitutively activated STAT3 (Fig. 6).

## Challenges and future outlook

The utilization of peptide as a targeting could bring forth multiple advantages - such as highly specific, naturally degradable, easily synthesized, and simple tunability with a variety of linker chemistries, and potentially reduce side effects and toxicity (Wang et al., 2017). However, there are also various hurdles that needed to be overcome in order for these peptides to be developed in the clinics.

### Increasing peptide avidity

The affinity of a peptide is used to describe the strength of a peptide-ligand interaction. Most peptides possess high affinity towards target (nanomolar to micromolar), which could be considered as high affinity. However, a short, singular linear ligand, peptides usually lack avidity, that is the ability to bind to the target via multiple interactions that can synergize their binding to enhance the affinity and also lead to enhancement of target residence time resulting in high local concentration of the targeted molecules (Vauquelin and Charlton, 2013). To overcome this barrier, most researchers decorated short linear peptides on the surface of nanocarriers to increase the probability of the peptide to interact with the specific ligands. Rouslahti et al. fused NGR peptide to TNFα, a highly toxic cytokine whose clinical application was limited due to its systemic toxicity. These targeted cytokines were effective even at 1,000-fold lower concentration that than usual dose, therefore diminishing the highly toxic side effects of TNFα. The success of this peptide-cytokine fusion could be attributed to the fact that the quaternary structure of TNFα is a trimer and the NGR peptide could be attached to each subunit, resulting in three NGR peptide: TNFα ratio; enhancing receptor binding of NGR peptide through an avidity effect (Ruoslahti, 2012). Similar strategy was adopted by Jeon et al., when they described an EDB-targeting aptide fused to mouse TNF-α (mTNFα-APT_EDB_) for systemic and targeted therapy of EDB-overexpressing fibrosarcoma (Jeon et al., 2017). mTNFα-APT_EDB_ showed enhanced tumor inhibition properties than mTNFα alone or mTNFα linked to a nonrelevant aptide, without causing an appreciable toxicity as measured in body weight loss.

### Reducing peptide aggregation and increasing peptide solubility

Peptide with 5 amino acids and less are usually water soluble and their solubility decreases with the length of the peptide. However, peptides screened through phage display biopanning ranged between 7–30 amino acids. In aqueous solution, these peptides could be conforming to a specific 3-dimentional structure that allowed specific binding with their receptors. In practice, solubilizing peptides could be challenging as improper solubilization could result in the loss of the peptide activity. For this reason, Xiao et al. conjugated betaine onto bacterial xanthine guanine phosphoribosyltransferase (CG-GPRT) protein and the HIV inhibitory peptide (CG-T20). Results indicated that betaine could successfully reduce the protein/peptide aggregation and increased the solubility of both the protein and the peptide (Xiao et al., 2008), therefore suggesting that betaine conjugation could be used for reducing peptide aggregation and increasing peptide solubility.

### Overcoming poor cell permeability and increase cellular uptake

Since the discovery of natural CPPs (Tat and Penetratin), a number of synthetic peptides have since been added to this family; including short peptides comprising positive-charged amino acids such as arginine, lysine or histidine. To date, many reports on CPPs in their application as intracellular delivery vehicles, including small-molecule drugs (Lindgren et al., 2006), liposomes (Zhang et al., 2013), and biopharmaceuticals including oligonucleotides (Margus et al., 2012), peptides and proteins (Morris et al., 2001). When conjugated with TAT peptide, pro-apoptotic peptides (KLAKLAK)_2_ conjugate was taken up efficiently by mouse melanoma and human breast cancer cells in vitro. In the cells, the peptide conjugate further activated the endogenous caspase-3 which then cleaved the peptide resulting in release of the pro-apoptotic peptide (KLAKLAK)_2_. Not only this peptide induced apoptosis in these cells *in vitro,* they also inhibited the growth of mouse melanoma xenografts in mice (Kwon et al., 2008). This peptide conjugate also induced apoptosis in the various cancer cell lines such as melanoma, cervical carcinoma, non-small cell lung carcinoma, breast cancer (Yang et al., 2010).

Phage display biopanning technique has brought about an immense pool of high affinity and highly specific peptide ligand for solid tumor therapy. Many of these peptide-based targeting ligands have shown promising results in enhancing solid tumors therapy, including increasing tumor accumulation, highly specific tumor targeting and enhanced tumor inhibition effect when used in combination with anti-cancer drugs or biologics. However, for successful translation into the clinics, peptide-targeting ligand should be optimized for their affinity, avidity, water-solubility and target specificity. With the advancement of technology, one could now use a combined primary phage display screening and a secondary computational optimization method to develop an optimal peptide for targeting any receptor of interest in the field of solid tumor therapy.
